# Flurbiprofen inhibits heme induced NLRP3 inflammasome in Berkeley sickle cell disease mice

**DOI:** 10.3389/fphar.2023.1123734

**Published:** 2023-04-26

**Authors:** Dilpreet Kour, Mehboob Ali, Parul Khajuria, Kuhu Sharma, Palash Ghosh, Sukhleen Kaur, Surbhi Mahajan, P. Ramajayan, Sonali S. Bharate, Subhash Bhardwaj, Sanghapal D. Sawant, D. Srinivasa Reddy, Ajay Kumar

**Affiliations:** ^1^ Pharmacology Division, CSIR-Indian Institute of Integrative Medicine, Jammu, India; ^2^ Academy of Scientific and Innovative Research, Ghaziabaad, India; ^3^ Department of Pathology, Government Medical College, Jammu, India; ^4^ Shobhaben Pratapbhai Patel School of Pharmacy and Technology Management, SVKM’s NMIMS, Mumbai, India; ^5^ Natural Products and Medicinal Chemistry Division, CSIR-Indian Institute of Integrative Medicine, Jammu, Jammu and Kashmir, India

**Keywords:** sickle cell disease, flurbiprofen, NLRP3 inflammasome, inflammation, pain, Berkeley mice

## Abstract

Sickle cell disease (SCD) is accompanied by several complications, which emanate from the sickling of erythrocytes due to a point mutation in the *β*-globin chain of hemoglobin. Sickled erythrocytes are unable to move smoothly through small blood capillaries and therefore, cause vaso occlusion and severe pain. Apart from pain, continuous lysis of fragile sickled erythrocytes leads to the release of heme, which is a strong activator of the NLRP3 inflammasome, thus producing chronic inflammation in sickle cell disease. In this study, we identified flurbiprofen among other COX-2 inhibitors to be a potent inhibitor of heme-induced NLRP3 inflammasome. We found that apart from being a nociceptive agent, flurbiprofen exerts a strong anti-inflammatory effect by suppressing NF-κB signaling, which was evidenced by reduced levels of TNF-*α* and IL-6 in wild-type and sickle cell disease Berkeley mice models. Our data further demonstrated the protective effect of flurbiprofen on liver, lungs, and spleen in Berkeley mice. The current sickle cell disease pain management regime relies mainly on opiate drugs, which is accompanied by several side effects without modifying the sickle cell disease-related pathology. Considering the potent role of flurbiprofen in inhibiting NLRP3 inflammasome and other inflammatory cytokines in sickle cell disease, our data suggests that it can be explored further for better sickle cell disease pain management along with the possibility of disease modification.

## Introduction

Sickle cell anemia is a highly painful genetic disease. It originates due to a point mutation of A>T leading to the substitution of hydrophilic glutamic acid with hydrophobic valine in the beta chain of hemoglobin (Kato et al., 2018). This abnormal hemoglobin then leads to the formation of rigid and sickle-shaped red blood cells (RBCs) (Nader et al., 2020). Vasculopathy, vaso-occlusion, ischemia-reperfusion injury, hemolysis, inflammation, organ damage, and neuropathy are the attributive features of sickle cell disease (SCD) and major contributors to chronic pain (Hebbel et al., 2004; Kassim and DeBaun, 2013). Like other hemolytic diseases, hemolysis in SCD leads to the release of free heme extracellularly, which acts as a DAMP (damage-associated molecular pattern) to activate the NLRP3 inflammasome. Heme and sickle RBCs stimulate toll-like receptors (TLRs) and nod-like receptors (NLRs) and are known to induce inflammation in SCD (Belcher et al., 2014; Gladwin and Ofori-Acquah, 2014; van Beers et al., 2015; Pitanga et al., 2016). NLRP3 levels are upregulated in SCD and under pain crisis, the expressions are further escalated thereby exacerbating the condition (Vogel et al., 2018).

NLRP3 inflammasome is the vastly studied type of inflammasome due to its inclusion in several diseases and its ability to be activated by a wide range of stimulators (Yang et al., 2019). Inflammasomes are the cytosolic protein complexes comprising usually of a sensor protein, adaptor protein, and self-activating proteolytic enzyme; caspase-1 (Franchi et al., 2009). The formation of NLRP3 complex leads to activation of caspase-1 and cleavage of its downstream targets pro IL-1β and pro IL-18 and subsequently induces a caspase-dependent proinflammatory form of cell death; pyroptosis (Shi et al., 2015; Kesavardhana and Kanneganti, 2017). NLRP3 inflammasome is a prime mediator of chronic inflammation in various diseases, including SCD, and is a major contributor to the pathogenesis of these diseases (Ozaki et al., 2015; Kelley et al., 2019). Recently, it has been shown that hemolysis-induced fatality in various hemolytic diseases is mediated by activation of the NLRP3 inflammasome due to extracellular heme, through ROS production and K+ efflux from the cell (Dutra et al., 2014). Despite the major contribution of inflammation in SCD pathology, no drug targeting inflammation is yet approved. Though, there are few drug candidates in different phases of clinical trials.

**FIGURE 1:**
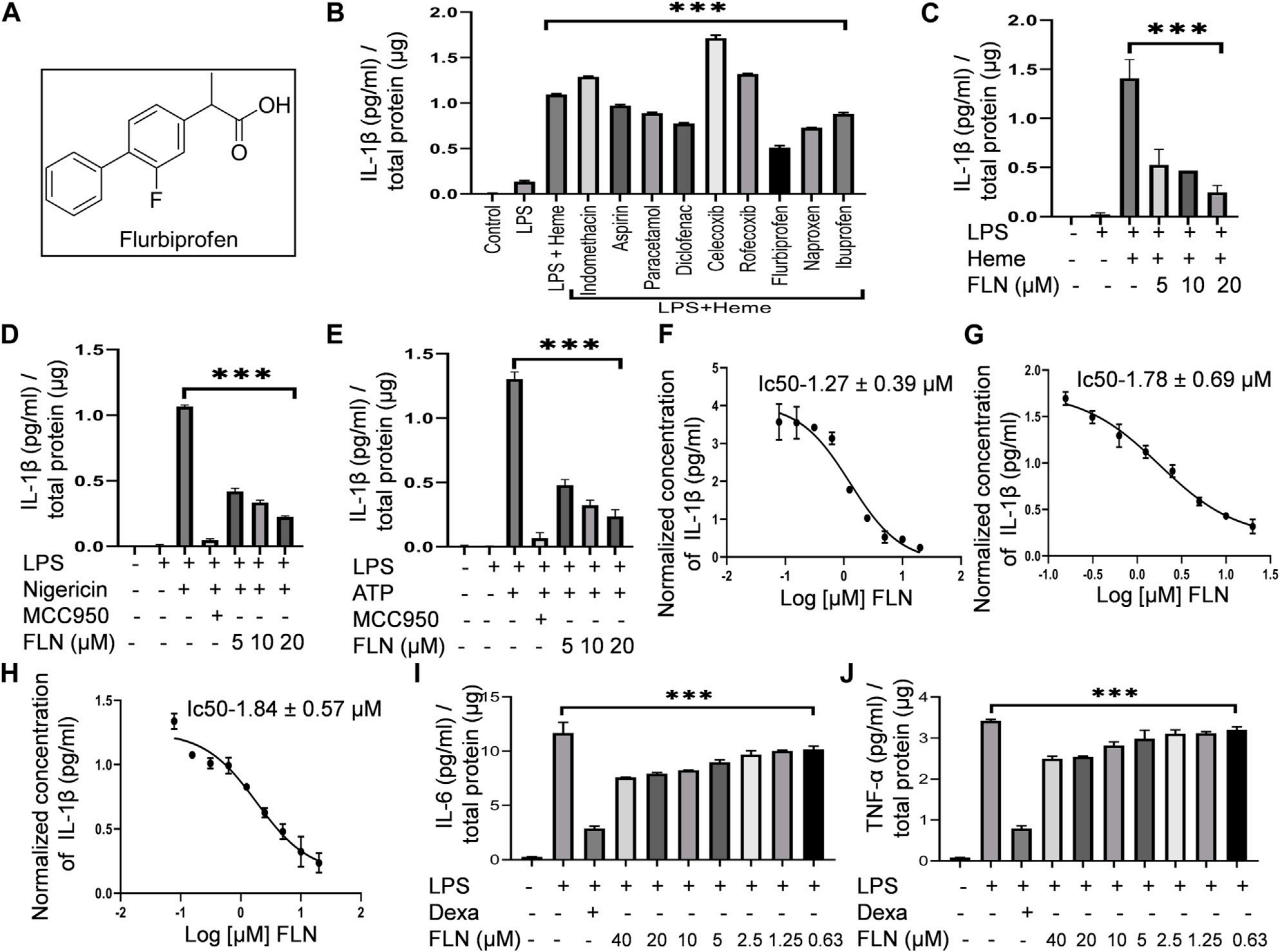
FLN (flurbiprofen) inhibits NLRP3 inflammasome activated against heme, Nigericin, and ATP by inhibiting IL-1β levels. Differentiated THP-1 cells were treated with LPS 100 ng/mL for 4 h. Drug treatment was given for 1 h at 10 μM concentration followed by 50 μM heme treatment for 1 h. For stimulation by ATP and Nigericin, J774A.1 cells were primed with LPS 1 μg/mL for 5.5 h followed by FLN or MCC950 (100 nM) treatment for 1 h. ATP at 3 mM for 30 min or Nigericin at 10 μM for 1 h was used as a secondary signal. **(A)** Structure of racemic mixture of Flurbiprofen. **(B)** Screening of NSAIDs against heme-induced NLRP3 inflammasome by detecting IL-1β level in the cellular supernatant. Analyzing IL-1β levels in the supernatant of **(C)** heme stimulated THP-1 cells and in **(D)** Nigericin and **(E)** ATP stimulated J774A.1 cells. IC50 of FLN for IL-1β against **(F)** heme induced THP-1 cells, and **(G)** Nigericin, and **(H)** ATP induced J774A.1 cells. FLN inhibits inflammation caused by IL-6 and TNF-α. J774A.1 cells were treated with 1 μg/mL LPS for 24 h following pretreatment of the drug or dexamethasone (10 μM) for 1 h. Levels of **(I)** IL-6 & **(J)** TNF-α were analyzed in supernatant through ELISA. MCC950 and Dexamethasone were taken as standard inhibitors. The data presented here is the mean of three independent experiments ±SD. Statistical significance was measured in GraphPad prism 8 by using one-way ANOVA and Bonferroni test as *post hoc*. *p* < 0.05 was taken as significant. *p* values ***p < 0.001, **p < 0.01, *p < 0.05.

In this study, we attempted to address two important aspects of SCD pathology that is inflammation and pain. For this purpose, we screened various NSAIDs (non-steroidal anti-inflammatory drugs) and identified flurbiprofen as one of the drugs, which inhibited heme-induced NLRP3 inflammasome and further improved the SCD pathology in the SCD transgenic mice, Berkeley model. Flurbiprofen is an FDA-approved drug for mitigating the symptoms like pain, swelling, and joint stiffness in osteoarthritis and rheumatoid arthritis (Figure 1A). It belongs to phenylalkanoic acid derivative family of NSAIDs (Brogden et al., 1979; Buchanan and Kassam, 1986; Richy et al., 2007). Flurbiprofen is a cyclo-oxygenase-2 (COX-2) inhibitor (Abdel-Aziz et al., 2012). Our data suggest that the nociceptive and anti-inflammatory properties of flurbiprofen can be further explored in human studies for better management of SCD pathology.

## Materials and methods

### Chemicals, reagents and antibodies

The details of all the chemicals, reagents and antibodies used in this manuscript have been given in the Supplementary Table S1 (ST-1).

### Cell culture

Human leukemia cell line THP-1 was obtained from ATCC. Mouse macrophage cell line J774A.1 was purchased from ECACC. THP-1 was grown in RPMI and J774A.1 was grown in DMEM, supplemented with 10% FBS, penicillin G and streptomycin. Cells were used within fixed passage ranging p18-p25 for J774A.1 and p2-p8 for THP-1 and maintained at 37°C in CO_2_ incubator.

### NLRP3 inflammasome activation

J774A.1 was seeded at a density of 0.4 × 10^6^ in 24 well plate for ELISA and at a density of 1 × 10^6^ in 6 well plate for western blotting. Cells were primed with LPS 1 μg/mL for 5.5 h and washed with incomplete media (media without serum) to remove any remnant serum particle. Flurbiprofen treatment and the second signal stimulus were given in incomplete media. Treatment of flurbiprofen was given for 1 h before the second stimulus of ATP or Nigericin. Freshly prepared ATP dissolved in 25% NaOH in water was added for 30 min at 3 mM concentration. Nigericin at 10 µM concentration was added for 1 h.

For heme treatment, THP-1 cells at 0.5 × 10^6^ density were seeded in 12 well plate for ELISA and at a density of 1 × 10^6^ in 6 well plate for western blotting. Monocytes-derived macrophages were obtained by treating THP-1 cells with 20 ng/mL phorbol 12-myristate 13- acetate (PMA) for 24 h. Differentiated cells after 48 h were primed with LPS 100 ng/mL for 4 h. Flurbiprofen treatment was given as described above. Heme prepared in 1 N NaOH and dissolved in RPMI was filtered and then added for 1 h at 50 µM concentration.

### ELISA

Experiments were terminated by collecting cell supernatant and adding lysis buffer (0.2 N NaCl and 1% Triton-X 100) to the cell pellet for total cellular protein measurement. The collected supernatant was subjected to ELISA to measure the level of released IL-1β, TNF-α and IL-6. ELISA was performed according to the manufacturer’s protocol, and absorbance was measured at 450 nm. The concentration of the total estimated cytokines level was normalized with total cell protein.

### IC50 calculation

IC50 of flurbiprofen against different stimuli was calculated by using GraphPad Prism 8 software using non-linear regression analysis model. Log (inhibitor) vs. response curve was plotted.

### Immunoblotting

To measure the level of cleaved IL-1β and CASP1 cellular supernatant was concentrated using strataclean resin as described by Gupta et al. (2021). Briefly, samples were kept on rotation for 1 h, followed by high-speed centrifugation at 4°C. The supernatant was discarded. 2X sample buffer was added to resin and samples were heated at 95°C for 10 min.

For total cell protein analyses, lysates were prepared using radioimmunoprecipitation assay (RIPA) buffer, substituted with sodium fluoride, sodium orthovanadate, phenyl methyl sulfonyl fluoride and 1% protease inhibitor cocktail. For analyzing the protein level in mice tissue, spleen, liver, and lung tissues were homogenized in the same RIPA buffer used above. Proteins were then separated by SDS-PAGE and transferred to the PVDF membrane. The proteins of interest were probed with corresponding primary antibodies under optimized conditions and further incubated with the HRP conjugated secondary antibody. Results were obtained in the Genaxy ChemiDoc imaging system (Make: Syngene, Maryland, USA; Model: G: BOX, XT-4). Band density was quantified using ImageJ software.

### Microscopy

Cells were seeded on coverslips. After treatment, cells were fixed using 4% paraformaldehyde (pH 7.2) for 15 min followed by permeabilization with 0.1% Triton-X 100. Blocking was done for 30 min followed by incubation with the primary antibody against ASC overnight at 4°C. The cells were then incubated with secondary antibody (Hilyte 488) for 1 h at room temperature. After antibodies’ incubation, cells were stained with 4,6-diamidino-2-phenylindole (DAPI) (1 μg/mL) for 10 min. Following staining, the cells were washed and mounted on slides using mounting media (PBS and glycerol at a ratio of 1:9). The images were taken in CQ1 high throughput imaging system.

### Animals and ethical clearance

BALB/c mice or Sickle cell transgenic mice *Hba* ^*tm1Paz*^ *Hbb* ^*tm1Tow*^Tg (HBA-HBBs) 41Paz/J (Jax:003,342), Berkeley model, weighing 22–25 g were used in all animal experiments. All SCD transgenic mice were genotyped for Hba^*tm1Paz(−/−)*^ Hbb^*tm1Tow(−/−)*^ and Tg (HBA-HBBs) 41Paz/J^(tg/tg)^ genes as per the protocol of Jackson Laboratory with standardized PCR and RT-PCR cyclic conditions. Animal were kept at 12-h light dark cycle at 25°C temperature and 50%–60% relative humidity. All experiments performed were ethically approved by Institutional Animal Ethics Committee (IAEC) of CSIR-IIIM, Jammu, under the IAEC number 241/79/8/2021. Animals were sacrificed by CO_2_ asphyxiation at the end of experiments.

### Peritoneal inflammation model

BALB/c female mice were randomly divided into groups of 5 animals. Different animal groups were used for the ATP-induced inflammation study and heme-induced inflammation study. Animals were intraperitoneally injected with different doses of flurbiprofen, dissolved in PBS for 30 min. LPS was administered intraperitoneally at 2 mg/kg concentration for 4 h. ATP was administered (0.5 mL, 100 mM) for 15 min and heme (0.5 mL, 5 μM) for 1 h. Peritoneal lavage was collected by injecting 3 mL of incomplete RPMI media into the peritoneum. Cytokines level were measured by performing ELISA of collected lavages.

### Air pouch model

Air pouch inflammation model was induced as described by Ali et al. (2021). Mice were randomly divided into groups of 5 animals. Sterile air was injected on the back of BALB/c mice subcutaneously to form the air pouch. On third day, sterile air was again injected to retain the air pouch. On sixth day, flurbiprofen or colchicine (1 mg/kg) for 30 min was administered into the air pouch. Triturated monosodium urate salt, suspended in sterile PBS (3 mg/mL) was then administered into the air pouch for 6 h. After complete treatment, incomplete RPMI was rinsed into the air pouch and collected for the measurement of cytokines by performing ELISA.

### Dosing in Berkeley mice

Mice were randomly sorted into four groups; two disease control and two treatment group, n = 5. Mice in treatment groups were given an oral dose of flurbiprofen, 10 mg/kg once a day, for 7 days. Control mice were given saline. After the end of treatment, mice were made to run on a rotarod for 2 h (Make; Ugo Basile, Acceler rota-rod for mice 7,650) to exhaust them and induce the disease-like conditions. Two groups, one control group and one treatment group were assessed 4 h post running, and the other two groups were assessed 24 h post running.

### Behavioral studies

To check the effect of flurbiprofen on Berkeley mice, OFT (open field test), hot plate (Make; Ugo Basile, model-DS37), and Randall Selitto (Make; Ugo Basile, model-19782) tests were performed. To check the effect of flurbiprofen on neuropathic pain, their response to mechanical stress was measured using the Randall Selitto method. The thermal latency of both groups was studied using the hot plate method. OFT was performed to assess locomotor activity in a white box (45 × 60 cm). Distance traveled, speed and mobile episodes were recorded through an automated video tracking system using ANY-maze software.

### Measurement of cytokines in serum of Berkeley mice

After the termination of the experiment, blood was collected from the retinal orbital plexus (ROP) of mice and kept for clotting, followed by centrifugation at 300 g for 10 min at 4°C and serum was separated. Levels of IL-1β, TNF-α and IL-6 were determined by ELISA as per the manufacturer’s protocol.

### Sickling measurement in Berkeley mice

Blood was collected from ROP of mice in CPDA-1 (Citrate Phosphate Dextrose solution with Adenine) buffer 2 h post running of mice on rotarod. To visualize RBCs at optimum density, blood was diluted in HBSS (Hanks’ Balanced Salt Solution). Diluted blood was added to 96 well plate and observed under the light microscope (Make: Magnus Opto Systems; Model: INVI) and images were taken at ×40 magnification. RBCs with round, disc-like morphology were considered normal, whereas sickle, star, or distorted-shaped RBCs were considered sickle. Number of normal and sickle RBCs were counted manually and percentage sickling was calculated using the formula; number of sickle cells/number of total cells ×100.

### Histopathology

The histopathological analysis of spleen, liver and lungs tissue were performed by staining tissues with hematoxylin-eosin (H & E) stain. Briefly, tissues were fixed using 10% Neutral buffered formalin solution. Fixed tissues were embedded in paraffin and then sectioned, dehydrated, and stained with H&E dye. The slides were observed at ×40 magnification under the light microscope (Make: Magnus Opto Systems; Model: INVI).

### Statistical analysis

Statistical analysis was done by applying one-way ANOVA test. Bonferroni test was used as a *post hoc* to measure statistical significance among multiple samples. The data values given here represent the mean of independent experiments ±SD. *p*-value <0.05 was considered significant. Data analysis and statistical significance were calculated by using GraphPad Prism 8 software.

## Results

### Flurbiprofen inhibited NLRP3 inflammasome activated by heme

With the information of studies reporting inhibition of NLRP3 inflammasome by certain NSAIDs like diclofenac sodium, flufenamic acid, meclofenamic acid, etc., we decided to screen more NSAIDs against NLRP3 inflammasome. However, with a focus of sickle cell disease, we screened these drugs against heme-induced NLRP3 inflammasome in THP-1 cells. Our data indicated that among all the screened NSAIDs, flurbiprofen at 10 µM showed the most potent activity (Figure 1B). Though, other drugs like naproxen, diclofenac sodium, ibuprofen, paracetamol, and aspirin also displayed mild inhibitory activity (Figure 1B). We further checked if flurbiprofen could also inhibit NLRP3 inflammasome induced by other stimulants like nigericin and ATP at three different concentrations that are 5, 10, and 20 µM. For heme-induced NLRP3 inflammasome, we used THP-1 cells whereas, for nigericin and ATP, we used J774A.1 cells. The results clearly showed that flurbiprofen exerts anti-NLRP3 activity against all the stimuli at these concentrations (Figures 1C–E). MCC950 (100 nM) was used as a standard inhibitor of NLRP3 inflammasome in case of induction by nigericin and ATP, however, it did not show any inhibition of heme induced inflammasome. The IC50 value of flurbiprofen against heme, nigericin, and ATP-induced NLRP3 inflammasome was calculated to be 1.27 ± 0.39, 1.78 ± 0.69, and 1.84 ± 0.57 µM, respectively (Figures 1F–H). Along with IL-1β, we checked the inhibitory effect of flurbiprofen against LPS induced inflammation in J774A.1 cells. Flurbiprofen significantly suppressed the levels of proinflammatory cytokines’ IL-6 and TNF-α (Figures 1I, J). Dexamethasone (10 μM) was taken as a standard inhibitor of LPS induced inflammation.

### Flurbiprofen suppressed cleavage of IL-1β by inhibiting NLRP3 mediated activation of CASP1

**FIGURE 2:**
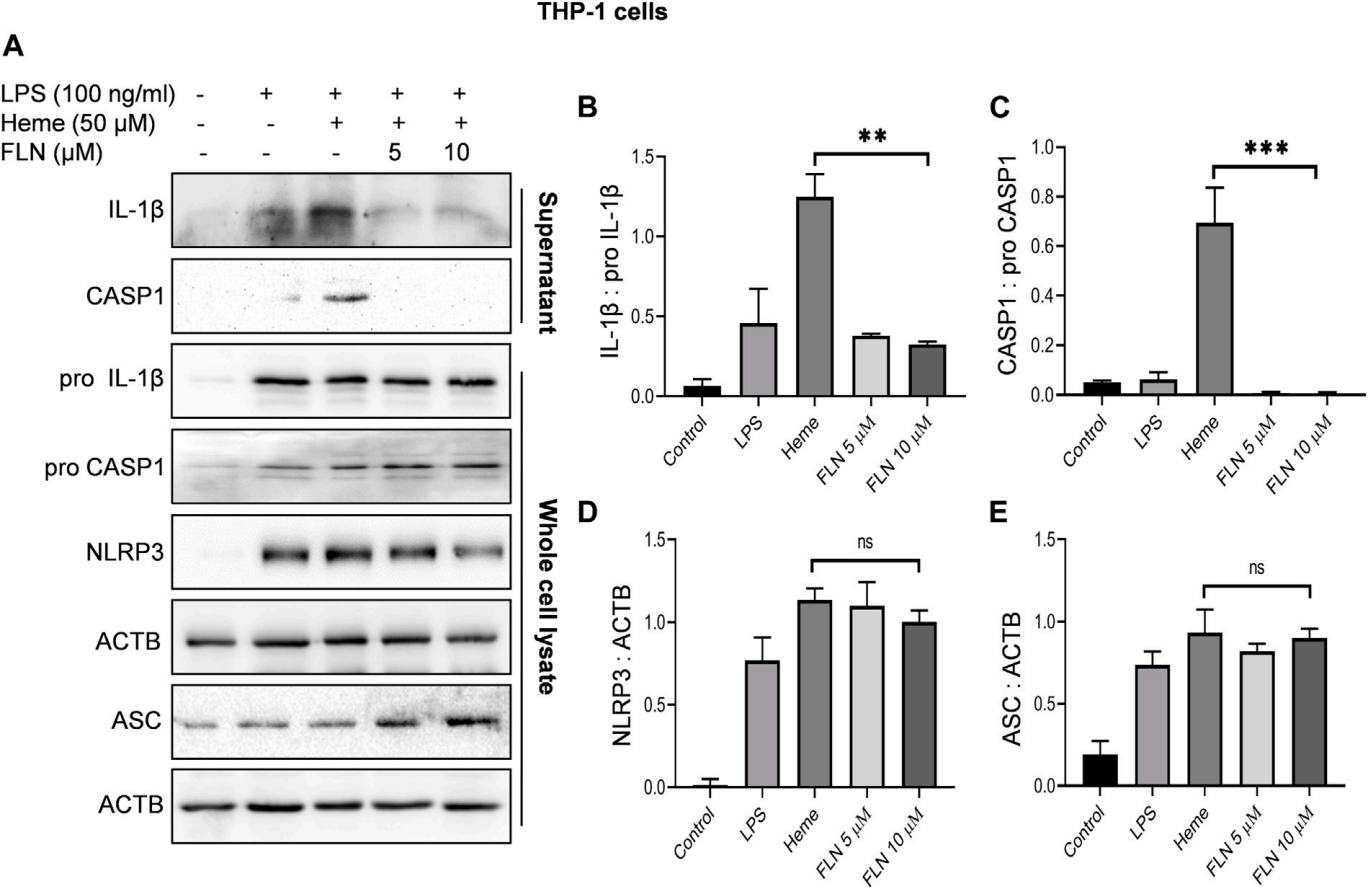
Effect of FLN on NLRP3 inflammasome complex proteins in heme induced THP-1 cells. Cells were treated as described in Figure 1. Levels of cleaved IL-1β and cleaved CASP1 were checked by concentrating supernatant proteins and analyzing it through immunoblotting. Levels of pro CASP1, pro IL-1β, NLRP3 and ASC were measured in whole cell lysates. **(A)** Immunoblots of NLRP3 inflammasome complex proteins. Densitometry analysis of immunoblots given in Figure 2A; **(B)** IL-1β, **(C)** CASP1, **(D)** NLRP3, and **(E)** ASC protein in THP-1. Cleaved proteins were normalized with their pro-forms. For NLRP3 and ASC, ACTB was used as an internal control. Band densities were quantified using Image J software. The data presented here is the mean of three independent experiments ±SD. Statistical significance was calculated using one-way ANOVA and Bonferroni test as *post hoc*. *p* < 0.05 was considered significant. ***p < 0.001, **p < 0.01, *p < 0.05.

**FIGURE 3:**
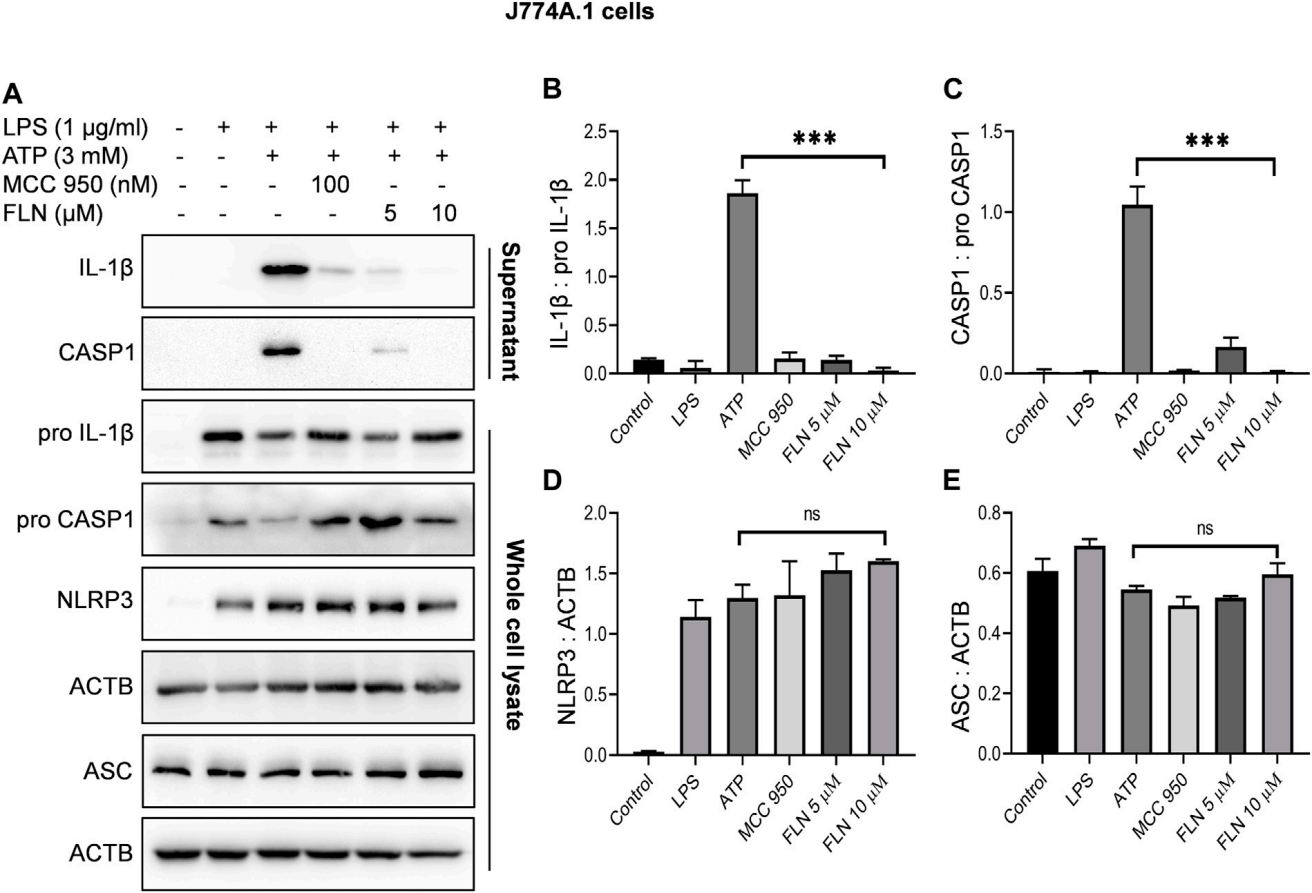
Effect of FLN on NLRP3 inflammasome complex proteins in ATP stimulated J774A.1 cells. Cells were treated as given in Figure 1. Cleaved IL-1β and cleaved CASP1 levels were analyzed in concentrated supernatant through immunoblotting. Levels of pro CASP1, pro IL-1β, NLRP3 and ASC were checked in whole cell lysates. **(A)** Immunoblots of NLRP3 inflammasome complex proteins. Densitometry analysis of immunoblots given in Figure 3A; **(B)** IL-1β, **(C)** CASP1, **(D)** NLRP3, and **(E)** ASC protein in J774A.1 cells. Cleaved proteins were normalized with their pro-forms. NLRP3 and ASC proteins were normalized with ACTB. Band densities were measured through Image J software. The data presented here is the mean of three independent experiments ±SD. Statistical analyses were done using one-way ANOVA and Bonferroni test as *post hoc*. *p* < 0.05 was considered significant. ***p < 0.001, **p < 0.01, *p < 0.05.

After confirming the inhibitory activity of flurbiprofen against NLRP3 inflammasome, we analyzed its effect on individual proteins constituting NLRP3 inflammasome complex in two different cell types. THP-1 cells were used to induce NLRP3 inflammasome by heme and J774A.1 cells were used for ATP dependent induction of inflammasome. Flurbiprofen at 5 and 10 µM significantly suppressed the cleavage of CASP1 and IL-1β as observed in the precipitated proteins from the supernatant of both the cell types (Figure 2; Figure 3). We also checked the levels of pro CASP1, pro IL-1β, ASC and NLRP3 in the whole cell lysates treated with flurbiprofen. The data indicated that the flurbiprofen did not have any effect on the levels of these proteins in both the cell types irrespective of the stimuli used for induction of NLRP3 inflammasome (Figures 2, 3).

### Flurbiprofen inhibited heme and ATP induced NLRP3 complex formation by preventing ASC oligomerization

**FIGURE 4:**
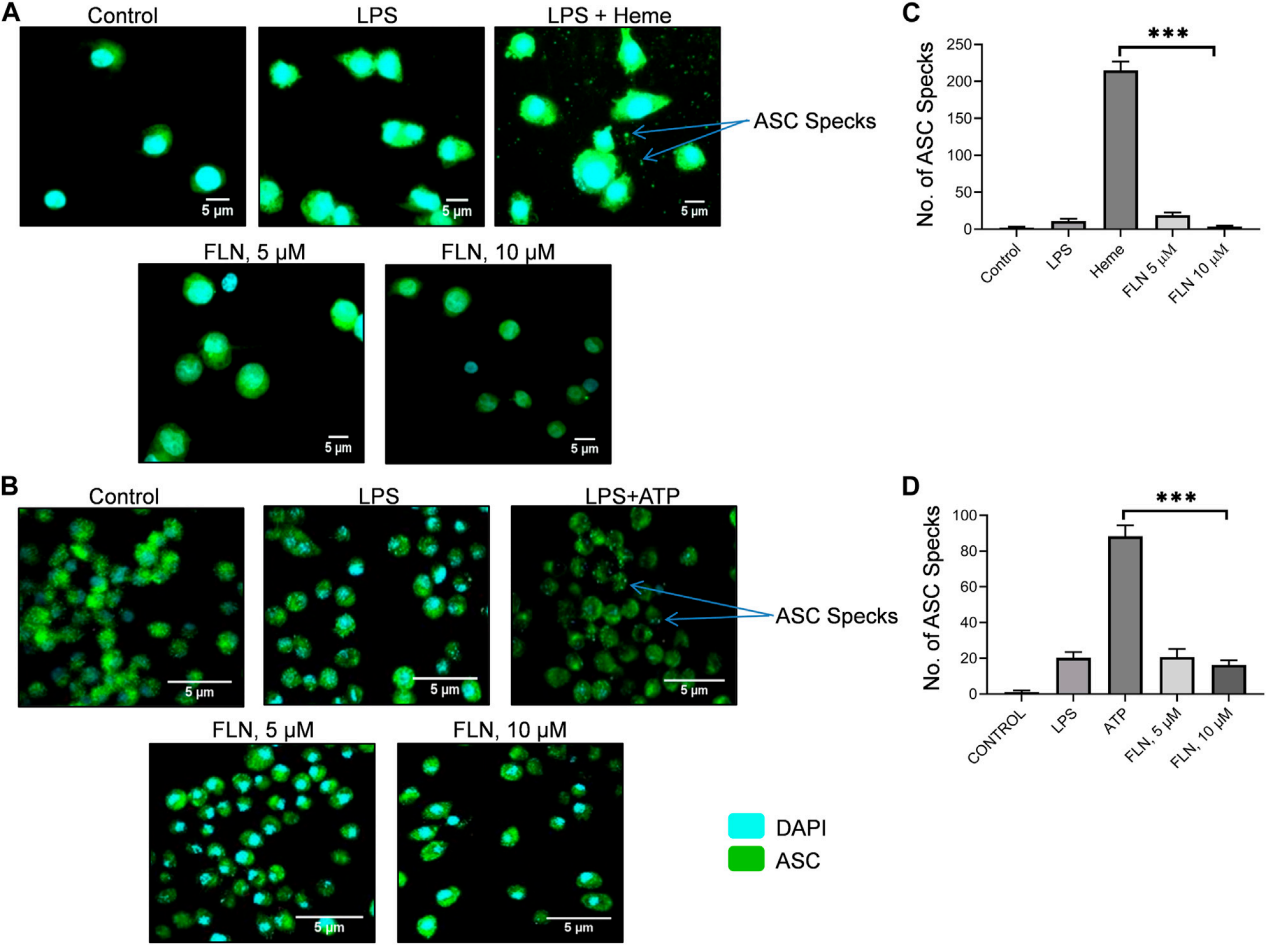
FLN inhibits ASC speck formation in THP-1 and J774A.1 cells. ASC specks were seen through CQ1 high throughput microscope. Scale bars were drawn in ImageJ software. ASC oligomerization or specks are visualized as green dots. Representative images of ASC specks in **(A)** heme stimulated THP-1 cells **(B)** and in ATP stimulated J774A.1 cells. **(C)** number of specks quantified in THP-1 cells. **(D)** number of specks quantified in J774A.1 cells. Statistical significance of mean data presented (n = 3) ± SD was seen by applying one-way ANOVA analysis and *post hoc* Bonferroni test. P values ***p < 0.001, **p < 0.01, *p < 0.05 Full images of data presented in Figure 4 are given in Supplementary Figures S1A, S1B.

The inhibition of cleavage of CASP1 without affecting the levels of proteins involved in NLRP3 inflammasome formation indicated that flurbiprofen might be inhibiting the NLRP3 inflammasome complex formation. ASC oligomerization is a crucial step in the formation and activation of the NLRP3 inflammasome complex. Therefore, we induced NLRP3 in THP-1 cells by using heme, pretreated with 5 and 10 µM of flurbiprofen for 1 h. The cells treated with heme displayed a large number of specks indicating the oligomerization of ASC. However, in the cells pre-treated with flurbiprofen, the number of ASC specks was reduced by more than 50 folds at 10 µM (Figures 4A, C and Supplementary Figure S1A). Further, the J774A.1 cells, which were stimulated by ATP also showed a highly raised number of ASC specks, which were reduced significantly by 5 folds in cells treated with flurbiprofen (Figures 4B, D; Supplementary Figure S1B).

### Flurbiprofen reduced the NLRP3 mediated release of IL-1β and proinflammatory cytokines TNF-α and IL-6 in the peritoneal and air pouch models of inflammation

**FIGURE 5:**
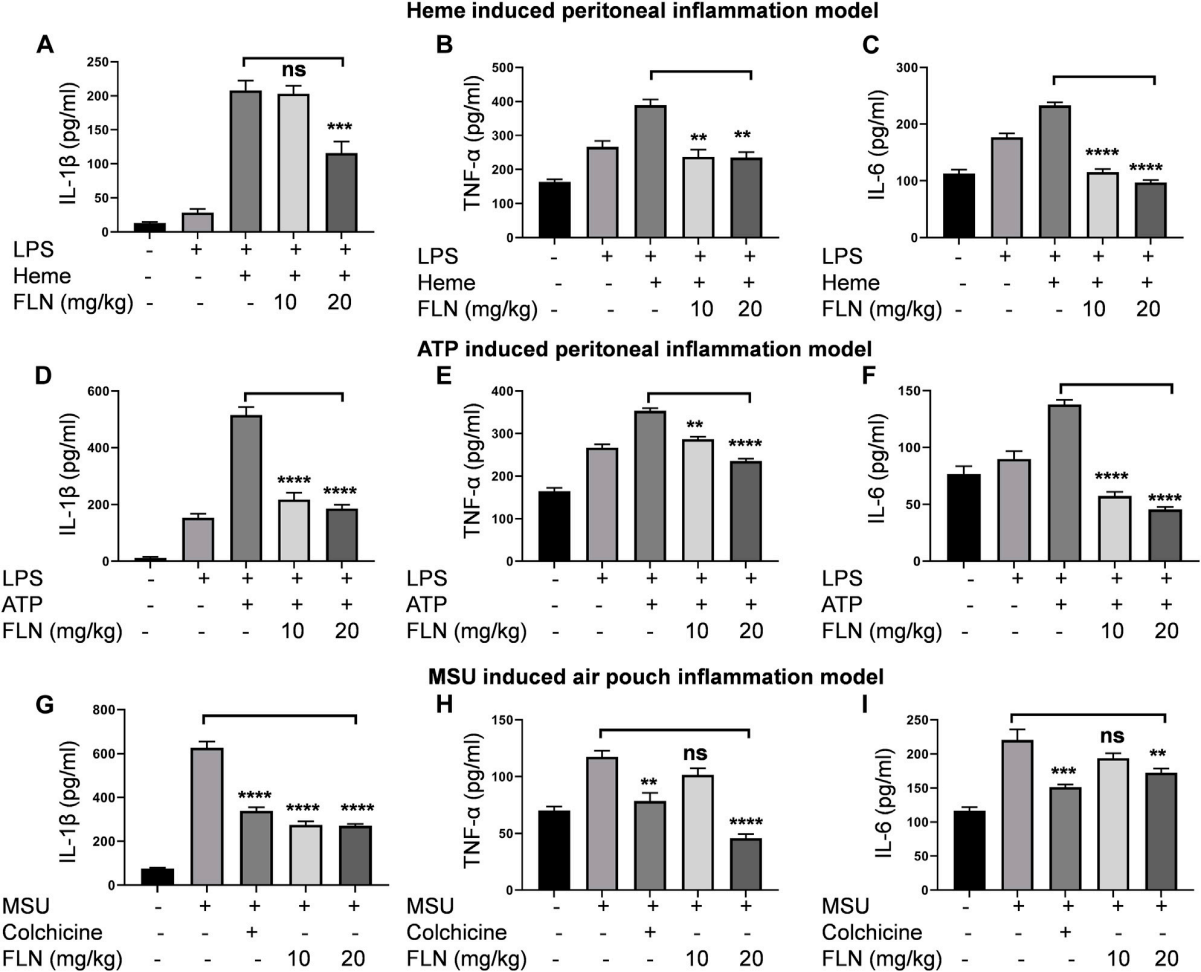
FLN inhibits proinflammatory cytokines production in different mice models. Levels of IL-1β, TNF-α, and IL-6 were analyzed in peritoneal or air pouch lavages of BALB/c mice through ELISA. BALB/c mice (n = 5) were injected with FLN 10 mg/kg and 20 mg/kg or vehicle for 30 min in the peritoneal cavity. LPS, 2 mg/kg was injected as the primary signal for inflammasome activation for 4 h, followed by heme (5 μM) for 1 h or ATP (100 mM) for 15 min. For MSU induced air pouch inflammation model, different doses of FLN, 10 mg/kg and 20 mg/kg, colchicine (1 mg/kg) or vehicle were injected into the air pouch in the BALB/c mice (n = 5) for 30 min followed by MSU (3 mg/mL) injection for 6 h. The levels of **(A)** IL-1β, **(B)** TNF-α and **(C)** IL-6 secreted in response to heme induced NLRP3 inflammasome and **(D)** IL-1β, **(E)** TNF-α and **(F)** IL-6 secreted in response to ATP induced NLRP3 inflammasome. The levels of **(G)** IL-1β, **(H)** TNF-α and **(I)** IL-6 secreted in MSU-derived air pouch inflammation in mice. Colchicine was used as a standard inhibitor in the air pouch model of inflammation. Data represent the mean of cytokines released by different mice in a group ±SD. Statistical significance was measured by one-way ANOVA followed by Bonferoni test as *post hoc*. *p* values ***p < 0.001, **p < 0.01, *p < 0.05.

After confirming the *in vitro* activity of flurbiprofen against NLRP3 inflammasome, we substantiated the anti-NLRP3 effect of flurbiprofen in three different mice models. In all the three models’ cleavage and release of IL-1β was considered as a marker of NLRP3 inflammasome activation. In the peritoneal inflammation models i.p. injection of LPS was used to prime the macrophages in the peritoneal cavity, which was followed by activation of the NLRP3 inflammasome by i.p. injection of second stimuli, which was either heme (Figures 5A–C) or ATP (Figures 5D–F). In both cases, treatment with flurbiprofen at 10 and 20 mg/kg of mice was given 30 min before priming with LPS. In the case of heme, flurbiprofen significantly suppressed the activation of NLRP3 inflammasome in the mice treated with 20 mg/kg (Figure 5A). However, the group, where ATP was used as a second signal displayed a highly significant suppression of NLRP3 inflammasome at both the dose of 10 and 20 mg/kg which was evident by the reduced release of IL-1β in the peritoneal lavage (Figure 5D).

Further, we used the air pouch model of MSU induced NLRP3 inflammasome to check the efficacy of flurbiprofen against another second stimulus. In this model, treatment with flurbiprofen at 10 and 20 mg/kg was given in the subcutaneous air pouch for 30 min, followed by MSU injection for 6 h. The mice injected with MSU alone showed highly raised levels of IL-1β in the lavage collected from the air pouch. However, mice treated with flurbiprofen showed significantly reduced levels of IL-1β, thus indicating the inhibition of MSU induced NLRP3 inflammasome (Figure 5G). The group treated with the standard drug colchicine at 1 mg/kg also showed similar results (Figure 5G). In all the three models, we also checked the levels of TNF-α and IL-6 to know the efficacy of flurbiprofen against these cytokines. The data revealed that mice injected with LPS alone expressed higher levels of TNF-α and IL-6, and moreover, the addition of the second stimuli, i.e., heme, ATP, and MSU further increased the levels of both these cytokines. Interestingly, flurbiprofen suppressed the levels of TNF-α (Figures 5B, E) and IL-6 (Figures 5C, F) in heme and ATP induced models at both the doses of 10 and 20 mg/kg. However, in the case of MSU induced model; flurbiprofen showed significant inhibition of TNF-α and IL-6 only at a higher dose of 20 mg/kg (Figures 5H, I).

### Treatment of sickle cell transgenic mice (Berkeley model) with flurbiprofen improved the pathophysiology related to SCD

**FIGURE 6:**
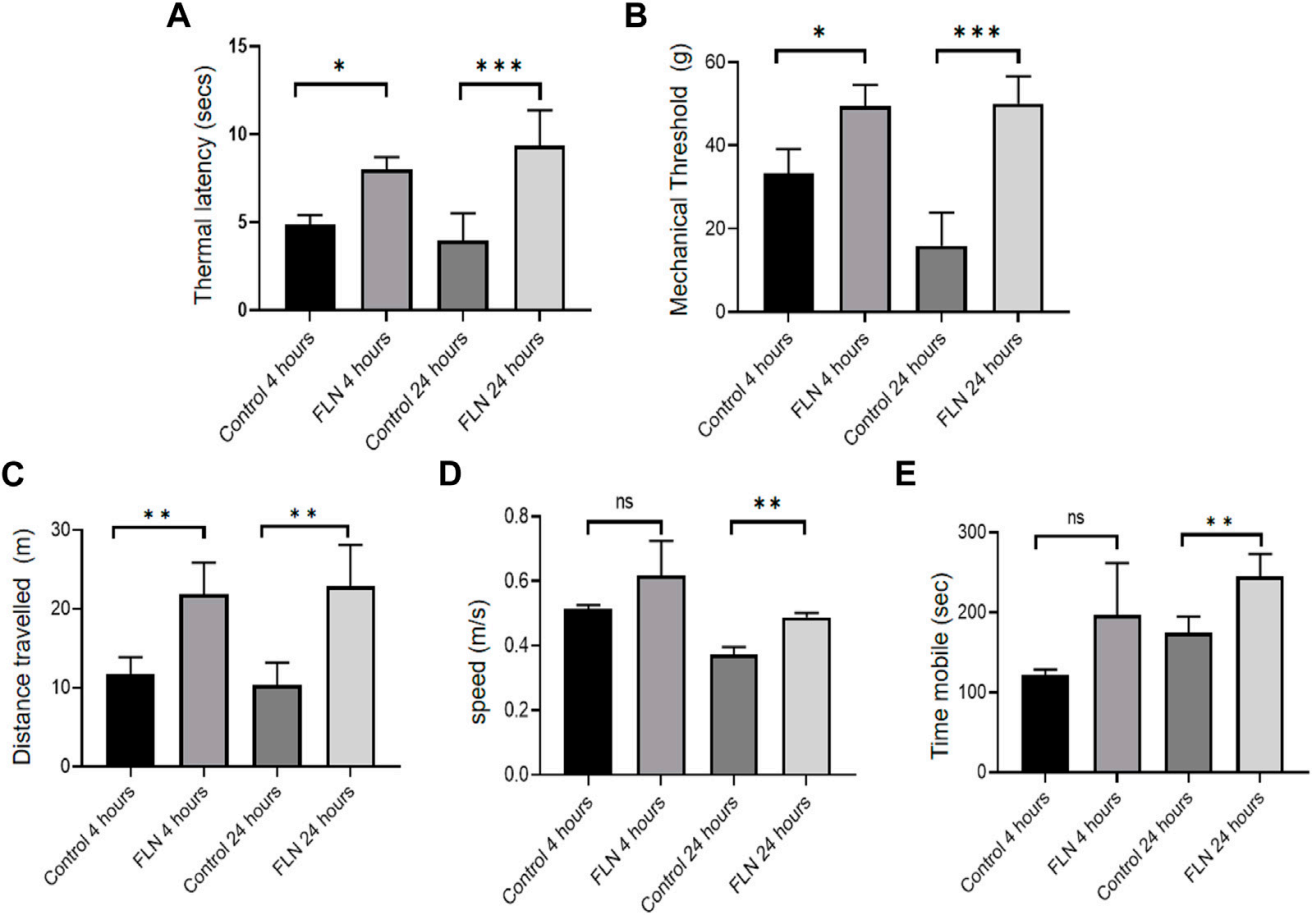
FLN ameliorates the pathophysiology of Berkeley mice. Berkeley mice (n = 5) were orally given FLN (10 mg/kg) or vehicle for 7 days. Post dosing, mice were made to run on the rotarod for 2 h to exhaust them and to induce stress. Pain behavior was checked 4 h and 24 h post running. FLN treatment resulted in an improved response to pain stimuli. **(A)** Thermal latency determined by hot plate method. **(B)** Mechanical threshold measured by Randall Selitto method. **(C)** Distance traveled by mice, **(D)** speed, and **(E)** mobility assessed by open field test and analyzed by ANYmaze software. Data represent the mean of responses by individual mice in a group ±SD. Statistical significance was checked by one-way ANOVA test and Bonferroni test as a *post hoc* test. *p* values ***p < 0.001, **p < 0.01, *p < 0.05.

The promising activity of flurbiprofen against heme induced NLRP3 inflammasome made us to test it in the transgenic mice model (Berkeley mice) of SCD for its effect on the pathophysiology of SCD. To induce the SCD pathophysiology, we standardized a protocol where exercise stress (by making them run on the rotarod for 2 h) could trigger sickling through hypoxia in Berkeley mice. We observed a rise in number of sickled RBC from 8.5% in control mice to about 40% in exercise stressed mice (Supplementary Figure S2). We treated the Berkeley mice (n = 5) with flurbiprofen (10 mg/kg) for 7 days before giving them exercise induced stress. Animals were given rest and were analyzed for pain behavior by using the hot plate method and Randall Selitto method at 4 and 24 h. The mice treated with flurbiprofen displayed significantly improved latency to thermal stimuli from 4.8 to 8 s at 4 h, which was improved further from 3.9 to 9.3 s at 24 h (Figure 6A). Berkeley mice treated with flurbiprofen also showed improvement against mechanical pain stimuli in the Randall Selitto test. The mechanical threshold of mice treated with flurbiprofen was increased from 33.3 to 49.4 g at 4 h, whereas, it was further improved from 15.8 to 50 g at 24 h (Figure 6B). The improved pain behavior was further reflected in their improved mobility, which was tested in the open field test. The mice treated with flurbiprofen covered more distances, 11.7–21.9 m after 4 h and 10.3–22.9 m after 24 h of exercise induced sickling (Figure 6C). The treated group also moved at a significantly higher speed as compared to the control group 0.37 m/s to 0.49 m/s at 24 h, respectively (Figure 6D). Further, the flurbiprofen group displayed more mobile time (245 s) when compared to the untreated control group (174 s) at 24 h. However, at 4 h the difference was not significant (Figures 6D, E).

### Treatment of SCD transgenic Berkeley mice with flurbiprofen reduced the level of inflammation

**FIGURE 7:**
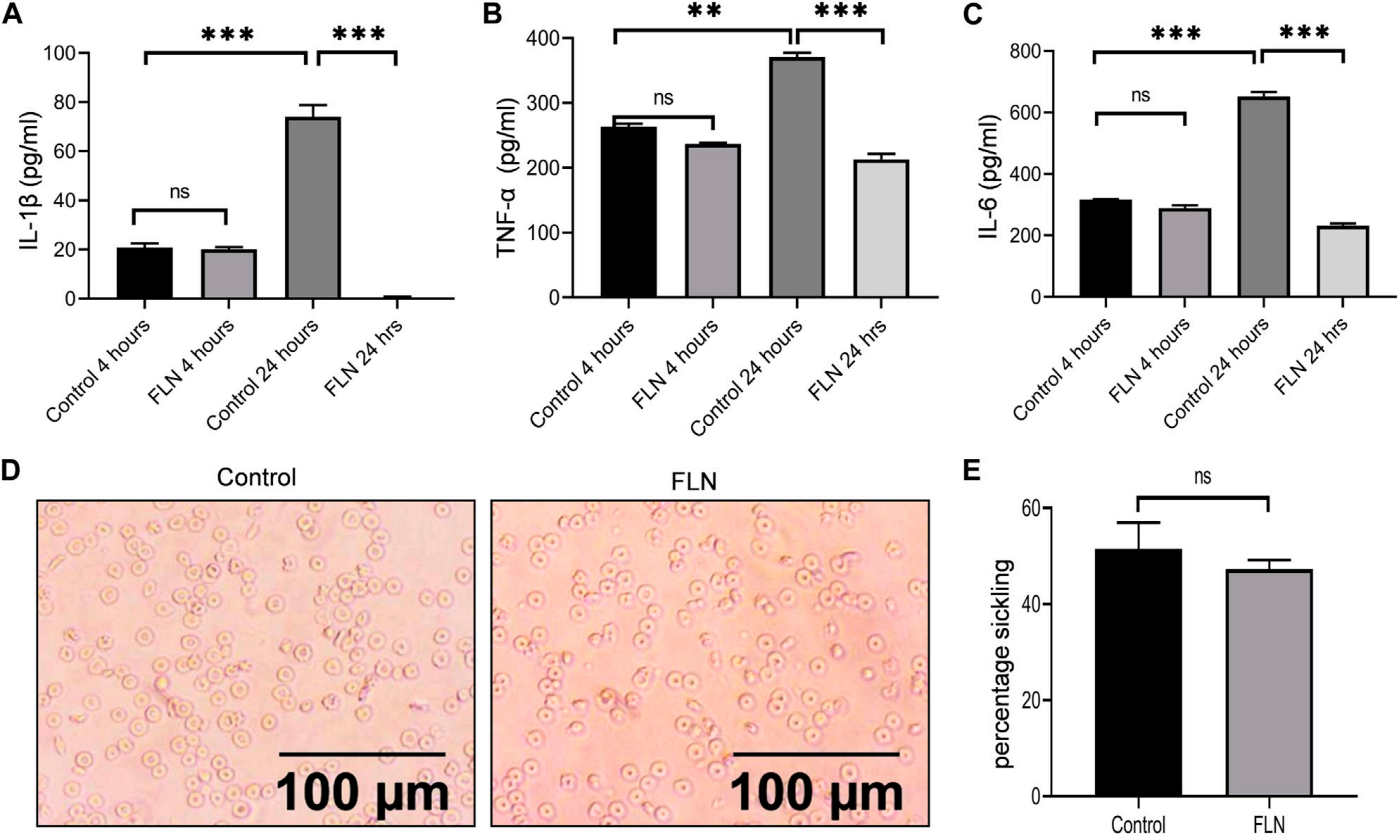
FLN alleviates the level of proinflammatory cytokine in Berkeley mice. Berkeley mice were treated as described in Figure 6. Post running, blood was collected through ROP and cytokine levels were analyzed in serum through ELISA. Effect of FLN treatment on **(A)** IL-1β, **(B)** TNF-α, and **(C)** IL-6 production in the serum of Berkeley mice. **(D)** Representative images of sickling in control mice and in FLN-treated mice blood. **(E)** Percentage sickling induced in the control group and FLN treated group. No. of sickle cells were counted manually and percentage sickling was calculated using the formula as number of sickle cells/total number of cells ×100. Statistical analyses were done using one-way ANOVA and Bonferroni test as *post hoc*. Significant data was considered where *p* < 0.05. *p* values ***p < 0.001, **p < 0.01, *p < 0.05.

After observing the significant improvement in the pain behavior post-treatment with flurbiprofen, we wanted to see if the anti-inflammatory behavior of flurbiprofen contributed to an overall improvement in the pathophysiology of SCD in Berkeley mice. Therefore, the blood samples were collected at two different time intervals (4 and 24 h) from the same groups of mice used in the pain behavior experiments for the analysis of inflammatory cytokines. The data clearly indicated that the mice treated with flurbiprofen had significantly low levels of IL-1β, TNF-α, and IL-6 in the blood samples collected after 24 h of exercise induced sickling in comparison with the control group (Figures 7A–C). However, the blood samples collected after 4 h of running the mice on rotarod displayed no significant difference between treated and untreated control groups (7A-C). The reduced level of inflammatory cytokines in flurbiprofen treated mice led us to check its effect on the sickling of RBCs. However, when we analyzed the level of sickling in the treated group, there was not any significant decline in the number of sickled erythrocytes when compared to the control group, implying no anti-sickling effect of flurbiprofen (Figures 7D, E).

### Flurbiprofen reduced inflammation in spleen, liver, and lungs of Berkeley mice

**FIGURE 8:**
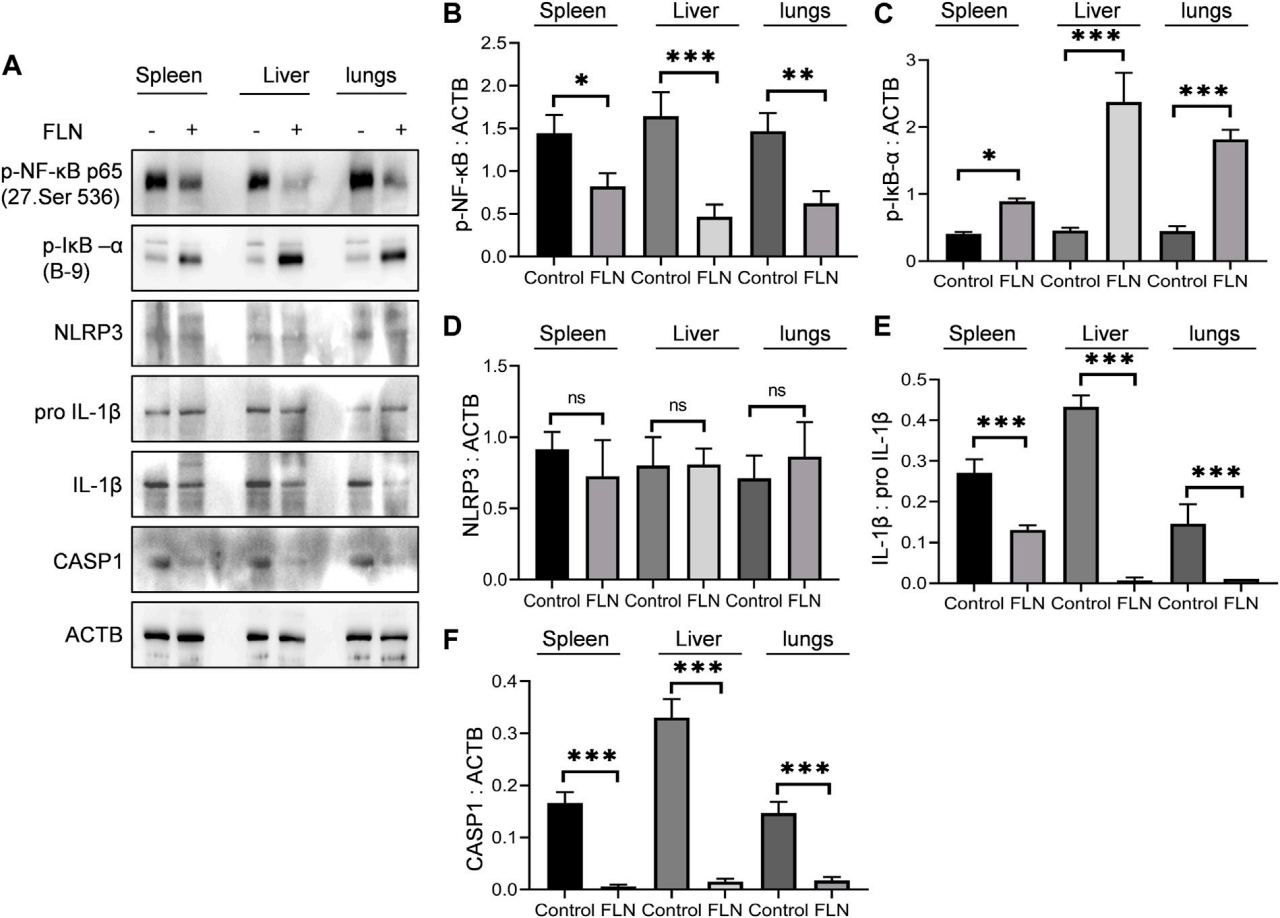
FLN protects the organs from inflammatory damage in SCD. Post-dosing of FLN in Berkeley mice, lysates were prepared by homogenizing tissues in RIPA buffer. **(A)** Immunoblots of inflammatory proteins in spleen, liver and lungs of Berkeley mice. Densitometry analysis of immunoblots given in Figure 8A; **(B)** p-NF-κB, **(C)** p-IκB-α, **(D)** NLRP3, **(E)** IL-1β, and **(F)** CASP1 measured using ImageJ software. ACTB was used as an internal control and for protein normalization. For cleaved IL-1β, pro IL-1β expression was used for normalization. One-way ANOVA analysis was done to check the statistical significance of data, and Bonferroni test was done for multiple comparisons. P< 0.05 was considered significant. *p* values ***p < 0.001, **p < 0.01, *p < 0.05.

Spleen, liver, and lungs undergo considerable damage due to sickled erythrocytes congestion, which produces an inflammatory environment in these organs. Therefore, to check the effect of flurbiprofen treatment, we isolated these organs from the mice used in the preceding experiment and checked the level of inflammatory proteins involved in NLRP3 inflammasome induction. All three organs in treated animals (n = 5) displayed significantly reduced levels of p-NF-κB (Ser 536), whereas the level of p-IκB (Ser 32) was significantly upregulated when compared to the control group (Figures 8A–F). However, the expression of proteins downstream of NF-κB including NLRP3 and pro IL-1β was not changed (Figures 8A–F). Further, flurbiprofen treatment led to a reduction in cleavage of NLRP3 inflammasome dependent proteins that are pro CASP1 and pro IL-1β (Figures 8A–F).

**FIGURE 9:**
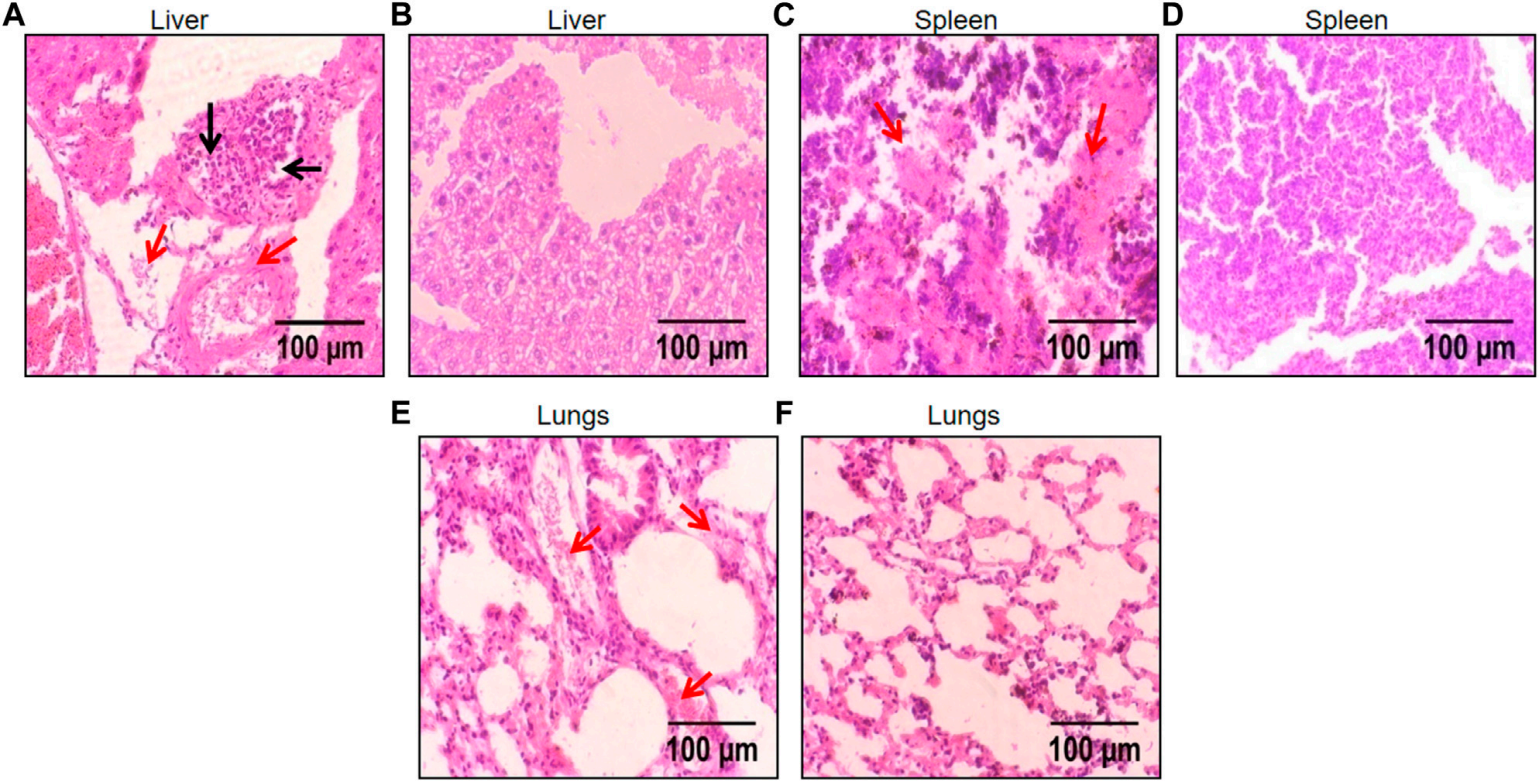
Histological images of liver, spleen, and lungs depicting protective effect of FLN in SCD mice. The representative H & E stained liver, spleen, and lungs tissue at ×40 magnification under the light microscope; **(A)** disease control liver **(B)** FLN treated liver **(C)** disease control spleen **(D)** FLN treated spleen **(E)** disease control lungs **(F)** FLN treated lungs. The black arrow indicates inflammatory cell infiltration and the red arrow indicates congestion by sickled RBCs.

The dwindling level of inflammation in these organs was also reflected during the histological analysis of these organs after H & E staining. A significant reduction in chronic infiltration of inflammatory cells and liver sequestration was observed in the liver tissue (Figures 9A–F). Whereas the spleen and lungs displayed highly reduced levels of congestion caused by sickled erythrocytes and improved tissue morphology (Figures 9A–F).

## Discussion

Inflammation and pain are crucial features of SCD pathology. Chronic upregulation of inflammatory cytokines such as IL-1β, TNF-α, IL-6, and IL-18 is usually found in the blood of SCD patients (Harlan, 1985; Pober, 1988; Francis and Haywood, 1992; Malave et al., 1993; Pathare et al., 2004). The release of heme from lysis of fragile sickled erythrocytes is primarily responsible for chronic inflammation in SCD (Chen et al., 2014). In the recent past, several studies have linked heme-induced NLRP3 inflammasome with SCD pathology. NLRP3 inflammasome activation has been shown to trigger platelet aggregation, thrombosis, vascular occlusion, and inflammation (Vogel et al., 2018). Therefore, NLRP3 inflammasome may be indirectly involved in the pain related with SCD. Additionally, pain is one of the main causes of hospitalization of SCD patients, which is usually managed by using opioid drugs (Brandow et al., 2020). Though, NSAIDs are also used to treat mild SCD pain (Brandow et al., 2020). With this information, we wanted to know if any of the NSAIDs can also be used to suppress heme induced NLRP3 inflammasome for better management of SCD.

Therefore, in this study, various approved NSAIDs were screened for inhibition of heme induced NLRP3 inflammasome and flurbiprofen was found to be the most promising amongst these drugs. Importantly, flurbiprofen was effective against NLRP3 inflammasome even when different types of second stimuli were used to activate NLRP3 inflammasome including ATP, nigericin and heme. Further, its anti-inflammatory activity was not restricted to NLRP3 inflammasome only as it also reduced the levels of TNF-α and IL-6 in various *in vitro* and *in vivo* models of inflammation.

NLRP3 inflammasome is a trimeric protein complex, which involves NLRP3, ASC, and pro caspase-1 (Kelley et al., 2019). The formation of complex leads to the self-activation of pro caspase-1 into its active proteolytic form that is caspase-1, which further cleaves pro IL-1β and pro IL-18 to their active forms (Franchi et al., 2009).

SCD pain is one of the severest forms of pain, which is often treated by opioid drugs. However, the chronic use of opiates can lead to several side effects, including constipation, tolerance, acute chest syndrome, renal toxicity, respiratory depression, addiction, etc. (Kopecky et al., 2004; Atici et al., 2005; Weber et al., 2008). Therefore, alternative and effective pain therapeutics such as COX-2 inhibitors may be considered for the management of SCD pain (Sadler and Stucky, 2019). COX-2 and NLRP3 inflammasome are related to each other as some of the studies suggest that COX-2 depletion may downregulate the NLRP3 inflammasome (Hua et al., 2015; Xue et al., 2019) Apart from that NLRP3 inflammasome can also activate COX-2/mPGES-1/PGE2 axis, which may further contribute to pain (Zhuang et al., 2017). A recent study by Iryna A. Khasabova et al. (2019) has suggested that systemic therapy with R-flurbiprofen can enhance the pain threshold and reduce the sensitivity to pain stimuli in Berkeley mice prostaglandin E 2 - glycerol (PGE2-G) by inhibiting its COX-2 mediated synthesis. This study highlighted the importance of PGE2-G in SCD related pain along with the effect of flurbiprofen on PGE2-G mediated pain in SCD. However, our data suggest that flurbiprofen may have an additional benefit of inhibiting NLRP3 mediated inflammation in SCD. Our data in wild-type mice validated our earlier findings that flurbiprofen is a strong inhibitor of heme induced NRLP3 inflammasome. These data were further confirmed in transgenic Berkeley mice, where NLRP3 inflammasome stimulated by exercise induced stress in mice was significantly inhibited along with other proinflammatory cytokines, TNF-α and IL-6. The activation of NLRP3 inflammasome in SCD transgenic mice evident by elevated IL-1β level in the serum 24 h post running mice on rotarod suggests that the sickling of RBCs caused by exercise in mice may have elevated the extracellular heme and HMGB1, which in turn activated the NLRP3 inflammasome (Chen et al., 2014; Xu et al., 2014). Chronic inflammation in SCD is strongly linked with pain and damage to several organs in SCD patients (Keleku-Lukwete et al., 2015; Zhang et al., 2015; Conran and Belcher, 2018). Behavioral analysis at 24 h displayed the decline in the neuropathic pain response by disease control mice when the circulating proinflammatory cytokines level increased in them. Moreover, the mice spent less time in mobile state after 24 h of exercise. However, the pain threshold level was observed to be significantly improved in flurbiprofen treated mice. Further, flurbiprofen treatment mitigated the pain after 4 h of running as well, when the inflammation was low in mice, suggesting this activity of flurbiprofen to ameliorate pain might be independent of its anti-inflammatory potency and depend more on already reported pain sensitizing pathways of flurbiprofen.

Furthermore, SCD pathology involves damage to various organs like the liver, spleen, and lungs, which is usually caused by vascular congestion and the infiltration of inflammatory cells into these organs (Keleku-Lukwete et al., 2015; Zhang et al., 2016; Fredman, 2019). Reduced levels of NLRP3 inflammasome mediated IL-1β in flurbiprofen treated mice organs is specifically important as chronic inflammation caused by IL-1β plays a crucial role in slowly damaging these organs (Wanderer, 2009; Lopez-Castejon and Brough, 2011).

In conclusion, our data suggest that flurbiprofen apart from having a nociceptive effect has a wide spectrum of anti-inflammatory activity, which encompasses inhibition of NLRP3 inflammasome and NF-κB dependent cytokines, TNF-α and IL-6. The prevalence of large-scale chronic inflammation in SCD pathology and role of NLRP3 in various pathological events like vascular occlusion, platelet aggregation, and thrombosis warrants the exploration of translating this data to human studies for better management of SCD.

## Figures and Tables

**Table:** 

**APS**	Ammonium persulfate
**ASC**	Apoptosis-associated speck-like protein containing a Caspase recruitment domain
**ATP**	Adenosine triphosphate
**CASP1**	Caspase-1
**COX-2**	Cyclo-oxygenase 2
**CPDA1**	Citrate phosphate dextrose adenine solution 1
**DAMPs**	Damage-associated molecular patterns
**DAPI**	4,6-diaminido-2-phenylindole
**ECL**	Enhanced chemiluminescence
**EDTA**	Ethylenediamine tetraacetic acid
**ELISA**	Enzyme-linked immunosorbent assay
**FDA**	Federal drug administration
**H&E**	Haematoxylin and Eosin
**HBSS**	Hanks balanced salt solution
**HEPES**	4-(2-hydroxyethyl)-1-piperazineethanesulfonic acid
**HRP**	Horseradish peroxidase
**IL-1β**	Interleukin-1β
**IL-6**	Interleukin-6
**LPS**	Lipopolysaccharide
**MSU**	Monosodium urate
**NLR**	NOD-like receptor protein 3
**NLRP3**	NLR family pyrin domain containing 3
**NSAID**	Non steroidal anti inflammatory drugs
**PMSF**	Phenylmethylsulfonyl fluoride
**PVDF**	Polyvinylidene difluoride
**RBC**	Red Blood Cell
**RIPA**	Radioimmunoprecipitation assay
**ROS**	Reactive oxygen species
**SCD**	Sickle Cell Disease
**SDS**	Sodium dodecyl sulfate
**SDS-PAGE**	Sodium dodecyl sulfate-polyacrylamide gel electrophoresis
**TEMED**	Tetramethylethylenediamine
**TLR**	Toll like receptor
**TNF-*α***	Tumor necrosis factor-*α*

## Data Availability

The raw data supporting the conclusion of this article will be made available by the authors, without undue reservation.
