# Supplementary material for: Flurbiprofen inhibits heme induced NLRP3 inflammasome in Berkeley sickle cell disease mice
Source: Front Pharmacol. 2023 Apr 26;14:1123734. doi: 10.3389/fphar.2023.1123734 (PMC10171431; doi:10.3389/fphar.2023.1123734)
Supplement: Supplementary file 1 [file Table1.DOCX]

**Flurbiprofen inhibits heme induced NLRP3 inflammasome in Berkeley sickle cell disease mice**

**Supplementary Figures:**

**FigureS1. FLN reduces the ASC specks formation.**


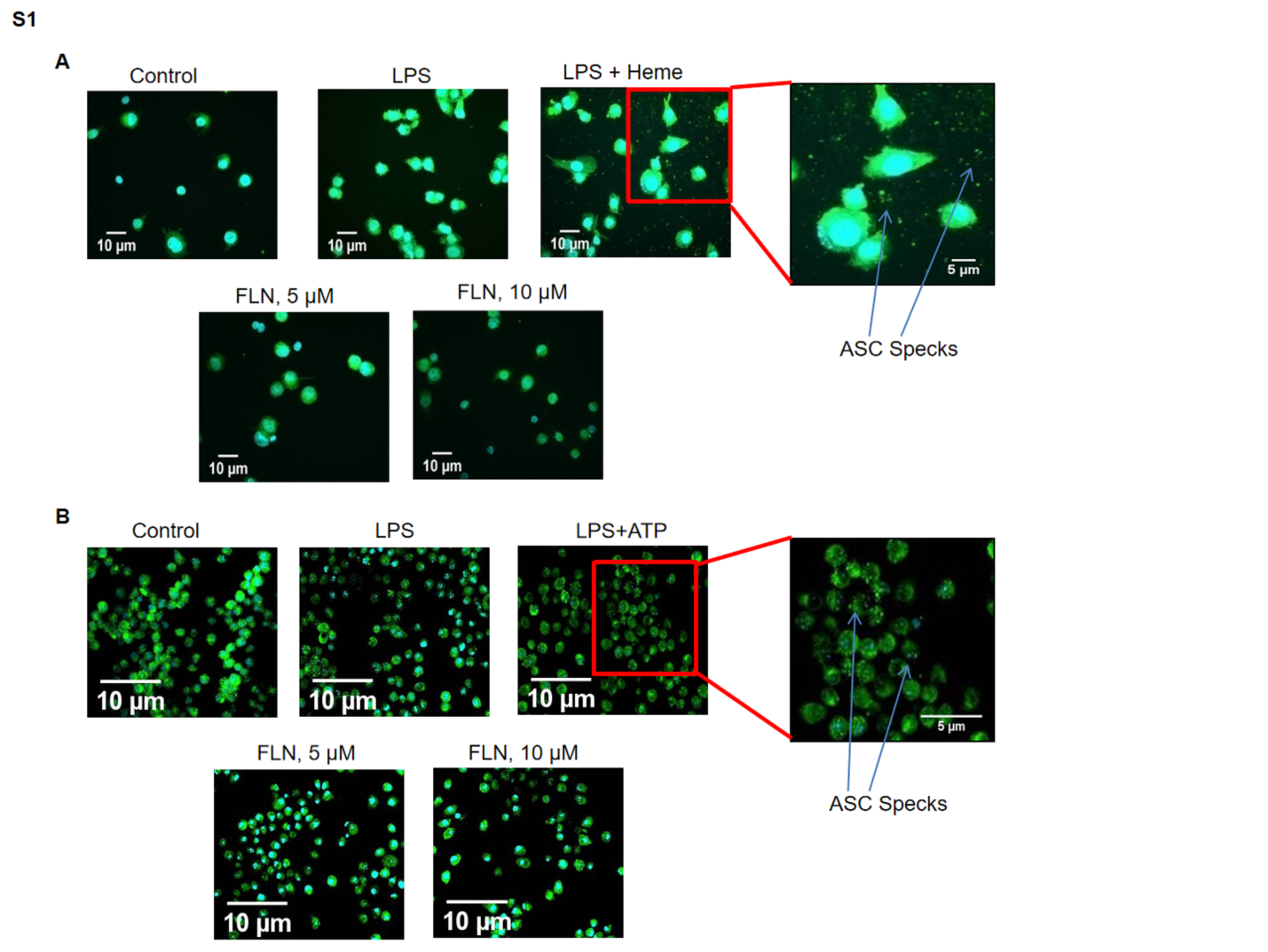


**Figure S1. FLN reduces the ASC specks formation.** Full figures of representative images given in **figure 4** are presented in figures **(S1A)** **and (S1B).** Blue fluorescence represents DAPI and green fluorescence is of ASC protein. Scale bars were drawn by image J software. Zoom-out images show ASC specks visualized as green dots.

**Figure S2. Exercise induces sickling in Berkeley mice**


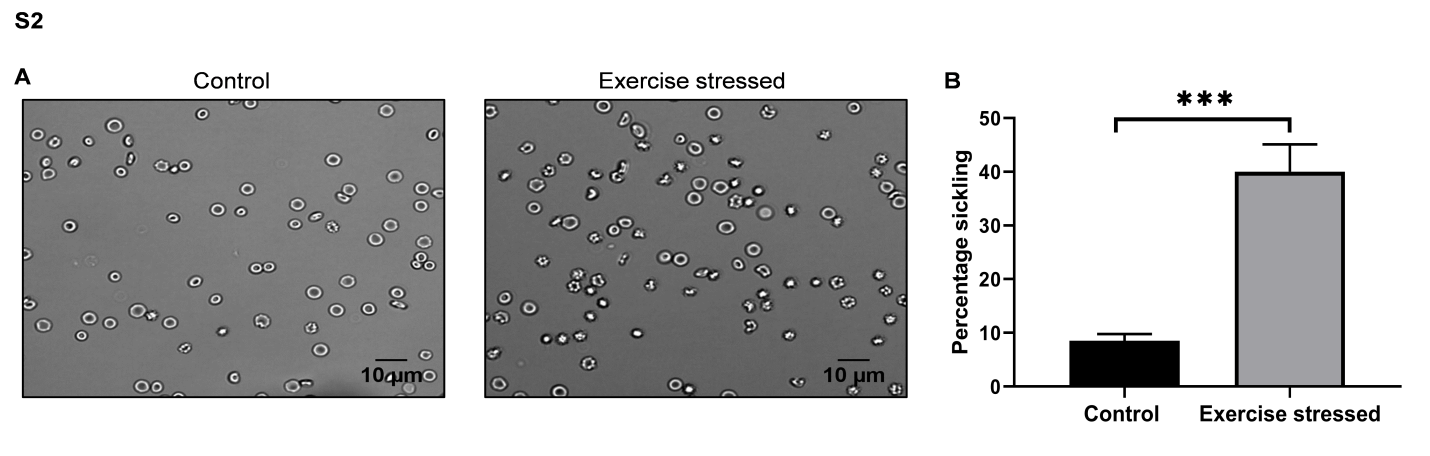


**Figure S2. Exercise induces sickling in Berkeley mice.** Blood from mice was collected in CPDA1 buffer before and after running them on rotarod for 2 h and inducing exercise derived stress. **(A)** Representative images depicting sickling of mice RBCs before and after exercise induced stress. **(B)** Graph representing percentage sickling in mice before and after exercise. Numbers of sickle cells were counted manually and percentage sickling was calculated using the formula; number of sickle cells/ total number of cells × 100. Data represent mean response of five mice in a group. Two tailed t-test was applied for analyzing the data. ***p < 0.001

**Table ST-1: List of chemicals, reagents and antibodies**

| Chemical | | Make | | Cat. No |
| --- | --- | --- | --- | --- |
| Flurbiprofen | | TCI Chemicals | | F0371 |
| Dulbecco’s modified eagle medium (DMEM) | | Sigma-Aldrich | | D1152 |
| RPMI-1640 | | Sigma-Aldrich | | R6504 |
| PMA | | Sigma-Aldrich | | P1585 |
| Streptomycin | | Sigma-Aldrich | | S6501 |
| Penicillin | | Sigma-Aldrich | | P3032 |
| Triton X-100 | | Sigma-Aldrich | | T8787 |
| Sodium bicarbonate | | Sigma-Aldrich | | S5761 |
| Phosphate buffer saline | | Sigma-Aldrich | | D5652 |
| HEPES | | Sigma-Aldrich | | H3375 |
| 4,6-diamidino-2-phenylindole(DAPI) | | Sigma-Aldrich | | D9542 |
| N,N′-methylene bisacrylamide | | Sigma-Aldrich | | M7279 |
| Sodium orthovanadate | | Sigma-Aldrich | | 450243 |
| Sodium fluoride | | Sigma-Aldrich | | 215309 |
| Tween 20 | | Sigma-Aldrich | | P7949 |
| Ammonium persulfate | | Sigma-Aldrich | | A3678 |
| TEMED | | Sigma-Aldrich | | T7024 |
| Glycerol | | Sigma-Aldrich | | G5516 |
| Hanks’ Balanced Salt Solution(HBSS) | | Sigma-Aldrich | | H6648 |
| Paraformaldehyde | | Sigma-Aldrich | | P6148 |
| Protease inhibitor cocktail | | Sigma-Aldrich | | P8340 |
| Heme | | Sigma-Aldrich | | 51280 |
| Uric acid sodium salt | | Sigma-Aldrich | | U2875 |
| Trizma-Base | | Sigma-Aldrich | | T6066 |
| SDS | | Sigma-Aldrich | | L3771 |
| Suberic acid bis(N-hydroxysuccinimide ester) | | Sigma-Aldrich | | S1885 |
| Lipopolysaccharide (LPS) | | Sigma-Aldrich | | L3129 |
| Nigericin sodium salt | | Sigma-Aldrich | | N7143 |
| ATP | | Sigma-Aldrich | | A6419 |
| EDTA | | Invitrogen | | 15575038 |
| Fetal Bovine Serum (FBS) | | GIBCO | | 10270106 |
| Acrylamide | | MP Biomedical | | 193982 |
| Glycine | | MP Biomedical | | 194825 |
| Albumin Bovine Fraction V | | MP Biomedical | | 160069 |
| Phenylmethylsulfonyl fluoride (PMSF) | | MP Biomedical | | 195381 |
| Skimmed milk | | Himedia | | GRM1254 |
| Sodium chloride | | Himedia | | MB023 |
| Sodium hydroxide | | Himedia | | MB095 |
| Strataclean resin | | Agilent | | 400714-61 |
|  | |  | |  |
| Antibodies | | | | |
| ASC | Santa Cruz Biotechnology | | SC-22514 | |
| CASP1 | Santa Cruz Biotechnology | | SC-56036 | |
| p-IκB-α (B-9) | Santa Cruz Biotechnology | | SC-8404 | |
| p-NFκB p65(27.Ser 536) | Santa Cruz Biotechnology | | SC-136548 | |
| HRP-linked anti-goat IgG | Santa Cruz Biotechnology | | SC-2354 | |
| NLRP3 | Cell signalling and technology | | 15101S | |
| anti-mouse IgG Alexa flour 488 | Cell signalling and technology | | 4412S | |
| HRP-linked rabbit IgG | Cell signalling and technology | | 7074S | |
| HRP-linked mouse IgG | Cell signalling and technology | | 7076S | |
| anti-mIL-1β | R & D biotechnology | | AF-401-NA | |
| anti-hIL-1β | R & D biotechnology | | AF-201-NA | |
| anti-ACTB | Sigma-Aldrich | | A3854 | |
|  |  | |  | |
| Kits and other reagents | | | | |
| PVDF Membrane | Millipore | | ISEQ00010 | |
| ECL-kit | Millipore | | WBKLS0500 | |
| Precision plus protein markers | Bio-Rad | | 161-0375 | |
| Bradford reagent | Bio-Rad | | 500-0006 | |
| Mouse IL- 1β ELISA kit | Invitrogen | | 88-7013-88 | |
| Mouse IL-6 ELISA kit | Invitrogen | | 88-7064-88 | |
| Mouse TNF-alpha ELISA kit | Invitrogen | | 88-7324-88 | |
| Human IL- 1β ELISA kit | BD Biosciences | | 557953 | |
